# Function-based selection of synthetic communities enables mechanistic microbiome studies

**DOI:** 10.1093/ismejo/wraf209

**Published:** 2025-09-17

**Authors:** Thomas C A Hitch, Johanna Bosch, Silvia Bolsega, Charlotte Deschamps, Lucie Etienne-Mesmin, Nicole Treichel, Stephanie Blanquet-Diot, Soeren Ocvirk, Marijana Basic, Thomas Clavel

**Affiliations:** Functional Microbiome Research Group, Institute of Medical Microbiology, University Hospital of RWTH Aachen, Pauwelsstrasse 30, Aachen, 52074, North Rhine-Westphalia, Germany; Functional Microbiome Research Group, Institute of Medical Microbiology, University Hospital of RWTH Aachen, Pauwelsstrasse 30, Aachen, 52074, North Rhine-Westphalia, Germany; Institute for Laboratory Animal Science, Hannover Medical School, Carl-Neuberg Str. 1, Hannover, 30625, Lower Saxony, Germany; UMR 454 MEDIS, Microbiologie Environnement Digestif et Santé, Université Clermont Auvergne, INRAE, UMR 454 MEDIS, 28 place Henri Dunant, F-63000 Clermont-Ferrand, France; UMR 454 MEDIS, Microbiologie Environnement Digestif et Santé, Université Clermont Auvergne, INRAE, UMR 454 MEDIS, 28 place Henri Dunant, F-63000 Clermont-Ferrand, France; Functional Microbiome Research Group, Institute of Medical Microbiology, University Hospital of RWTH Aachen, Pauwelsstrasse 30, Aachen, 52074, North Rhine-Westphalia, Germany; UMR 454 MEDIS, Microbiologie Environnement Digestif et Santé, Université Clermont Auvergne, INRAE, UMR 454 MEDIS, 28 place Henri Dunant, F-63000 Clermont-Ferrand, France; Gnotobiology Research Unit, German Institute of Human Nutrition Potsdam-Rehbruecke, Arthur-Scheunert-Allee 114-116, Nuthetal, 14558, Brandenburg, Germany; Research Group WESTGUT, ZIEL-Institute for Food & Health, Technical University of Munich, Gregor-Mendel-Str. 2, Freising, 85354, Bavaria, Germany; Institute for Laboratory Animal Science, Hannover Medical School, Carl-Neuberg Str. 1, Hannover, 30625, Lower Saxony, Germany; Functional Microbiome Research Group, Institute of Medical Microbiology, University Hospital of RWTH Aachen, Pauwelsstrasse 30, Aachen, 52074, North Rhine-Westphalia, Germany

**Keywords:** microbiomes, metagenomes, bioinformatics, synthetic communities, host–microbe interactions, inflammatory bowel disease

## Abstract

Understanding the complex interactions between microbes and their environment requires robust model systems such as synthetic communities (SynComs). We developed a functionally directed approach to generate SynComs by selecting strains that encode key functions identified in metagenomes. This approach enables the rapid construction of SynComs tailored to any ecosystem. To optimize community design, we implemented genome-scale metabolic models, providing *in silico* evidence for cooperative strain coexistence prior to experimental validation. Using this strategy, we designed multiple host-specific SynComs, including those for the rumen, mouse, and human microbiomes. By weighting functions differentially enriched in diseased versus healthy individuals, we constructed SynComs that capture complex host–microbe interactions. We designed an inflammatory bowel disease SynCom of 10 members that successfully induced colitis in gnotobiotic IL10^−/−^ mice, demonstrating the potential of this method to model disease-associated microbiomes. Our study establishes a framework for designing functionally representative SynComs of any microbial ecosystem, facilitating mechanistic study.

## Graphical Abstract

**Graphical Abstract:**
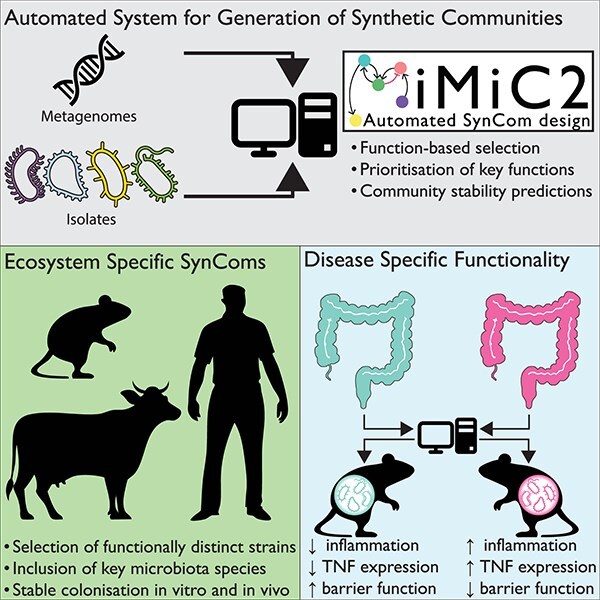

## Introduction

Microbiomes are complex communities of microorganisms, each of which contributes to the overall functionality of the ecosystem through its interactions with the other members. Microbiomes from the same habitat can vary greatly depending on their constituent species and functionality, many of which remain unknown, limiting our ability to mechanistically study the microbiota [1–3]. The variability in microbiomes can be accounted for by using synthetic communities (SynComs). SynComs consist of isolates, that functionally or taxonomically represent the ecosystem under study [4]. SynComs have been used experimentally to study the mammalian gut microbiota since the 1960s with the Schaedler flora, for which culturable and phylogenetically diverse members were selected. This ensured their accessibility and survival in the gut [5]. Since then, other SynComs have been developed to represent complex microbial communities in the mouse gut [6, 7], human gut [8–10], plant [11, 12], and even soil [13]. SynComs such as OMM12 and OMM19.1 have been designed to be broadly applicable and modular, allowing for the removal or addition of strains for mechanistic study of the gut ecosystem [14, 15]. More complex SynComs, such as hCom2, were designed to fill all potential niches by challenging a first iteration (hCom1) with human faecal samples to identify empty niches, and then filling these with available isolates to reach a total diversity of 119 members [10].

Identifying and including only the phylogenetically representative species in an ecosystem is a common method for creating a SynCom. However, this may exclude taxa that provide critical functionality. Therefore, the selection of members should prioritize function over taxonomy. Currently, only a few methods for SynCom design select members based on their functionality, and those that do prioritize a single function, rather than considering the landscape of functions that a complex community must perform [16, 17]. Although focused on single functions, the methods used could be modified to propose ecosystem representative SynComs. SynComs that represent an ecosystem should capture the functionality of the microbiota, and therefore fill the same ecological niches. This should include both general functions, such as carbohydrate utilization, but also ecosystem specific functions (i.e. seaweed utilization [18] or methanogenesis [19]). To capture the diverse functionality of a complete microbiota, SynComs should consist of multiple strains that can successfully co-occur within the ecosystem [20]. This can be achieved by studying taxonomic co-occurrence networks to identify interactions that support community stability [21]. To capture the functionality, taxonomic diversity, microbial interactions, and host–microbe interactions, SynComs consist of ~13 members on average [22]. Given the diverse range of microbiota that exist, automated tools are needed to facilitate the selection of members, yet only a few automated tools for SynCom design have been created [23, 24].

Our previous work developed a pipeline for the function-based selection of sample-specific SynComs [25]. This pipeline successfully selected distinct taxa for each ecosystem, but was unable to select a consensus SynCom representative of many samples from an ecosystem or capture critical functions known to be important. In this manuscript, we build upon this pipeline to ensure the prioritization and capturing of ecosystem critical functions, as well as selecting SynComs representative of groups of samples, rather than sample-specific SynCom design. By studying the consensus of functionality across multiple metagenomes, this approach provides representative SynComs that capture the functionality of a given ecosystem rather than an individual sample. Using this approach, we designed a SynCom based on samples from ulcerative colitis patients and confirmed its inflammatory impact on the host in a gnotobiotic mouse model.

## Materials and methods

### Metagenomic analysis

Metagenomic assemblies were either downloaded from published work [2, 26] or downloaded as raw data [2, 27, 28]. For samples downloaded as raw data, host reads were filtered using bbmap v2018 [29] with the following options; minratio = 0.9 maxindel = 3 bwr = 0.16 bw = 12 fast minhits = 2 qtrim = r trimq = 10 untrim kfilter = 25 maxsites = 1 k = 14 fast = t. The host-filtered reads were then assembled using MEGAHIT v1.2.9 [30] with the following options; —k-list 21,27,33,37,43,55,63,77,83,99 –min-count 5. The proteome of each metagenomic assembly were predicted using Prodigal v.2.6.3 [31] with the ‘-p meta’ option. Protein sequences were then annotated using hmmscan v.3.2.1 [32] with the gathering threshold ‘—cut_ga’ option against the Pfam database v32 [33], and outputted in tabular format.

### Genome collections

Genomes of either isolates (human = HiBC, mouse = miBC2, pig = PiBAC, rumen = Hungate1000) [7, 34–36], or metagenome-assembled genomes (MAGs) (human, global diversity) [2, 37] were downloaded and either taxonomically assigned using GTDB-Tk, or by converting their curated taxonomic assignments into a suitable format. The proteome of each genome was predicted using Prodigal v.2.6.3 [31]. Protein sequences were then annotated using hmmscan v.3.2.1 [32] with the gathering threshold ‘—cut_ga’ option against the Pfam database v32 [33], and outputted in tabular format. For each isolate genome, a metabolic model was generated using GapSeq v1.3.1 [38] using the ‘doall’ command and saved as an R object, which is compatible with BacArena. The default media selection was used for all models unless otherwise stated.

### Metabolic modelling

The BacArena toolkit [39] is used to conduct metabolic modelling due to its ease-of-install and compatibility with GapSeq generated metabolic models. This involves three premade scripts called: Single_Growth.R, Paired_Growth.R, and Combined_Growth.R. In each of these, an arena is made using the ‘Arena’ command (size: 100 × 100). The genome-scale metabolic model of each SynCom member is loaded into R and their default medium requirements included using ‘addDefaultMed’. This ensures that the media environment is not ecosystem specific, and hence the approach can be applied to all microbes and microbiota. Depending on the script, either a single SynCom member, a pair of members, or all members, will have 10 cells placed randomly within the arena for the single, paired, and combined scripts respectively. This is done with the ‘addOrg’ command. Growth is then simulated over 7 h using the ‘simEnv’ command. The limited simulation time may limit the inclusion and study of slow growing taxa. The growth of all modelled members is extracted and output in a comma-separated value (CSV) file for further analysis.

The Virtual Colon toolkit [40] was used with the default diet, diffusion rates, simulation sequences, colonic layers, colonocyte models, and placements. The Human Synthetic Community (HuSynCom) was simulated for 7 h and repeated 10 times to provide replicates [8].

### Synthetic community design

**Figure 1:**
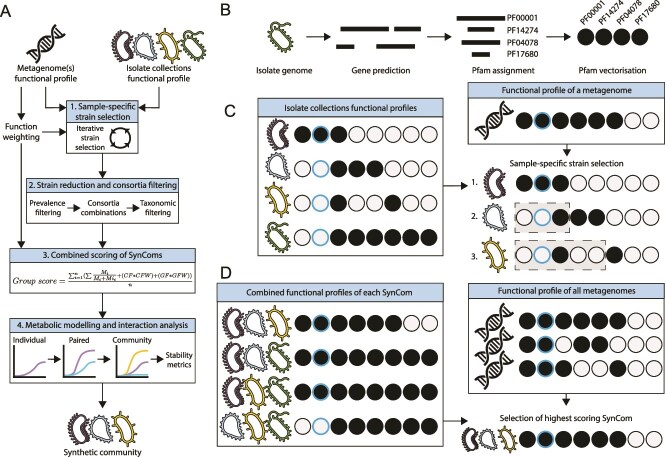
Workflow of function-based selection of SynComs. (A) Technical workflow of the steps involved in the selection of SynComs by MiMiC2, further details are provided in the methods. (B) Pfam vectorization of input genomes/metagenomes prior to SynCom selection. Each line represents a predicted protein sequence, which is then annotated to a Pfam, and finally the presence/absence of each Pfam within the input file is determined. The presence of a Pfam is indicated by a filled circle, an empty circle represents an absent Pfam. A blue border indicates that a Pfam is weighted and will be preferentially selected. (C) Simplified workflow for the sample specific selection of strains (step 1 in MiMiC2 workflow) to represent a single metagenome. On the left are the Pfam profiles of four strains, and on the right is the order each strain will be selected to represent the example metagenome profile. Dashed boxes cover the Pfams that are masked in the current iteration of selection due to having been covered by a previous strain. (D) Simplified workflow for the combined scoring of SynComs (step 3 in MiMiC2 workflow) to represent groups of metagenomes. On the left are the combined Pfam profiles of each consortium of strains, on the right are the Pfam profiles of each metagenome, and below is the highest scoring SynCom.

To facilitate the automated selection of SynComs based on the functionality in metagenomic samples, we created MiMiC2 based on our previously published work [25]. In this work, when discussing functions and functionality we are referring to the predicted Pfams encoded by an isolate, SynCom, or metagenome. For SynCom design, two binarized Pfam vectors are required; one for the input metagenome(s), and the second for the genome collection from which the members of the SynCom will be selected from (Fig. 1A and B). This can be generated by the ‘MiMiC2-butler.py’ script that accepts a folder containing hmmscan or hmmsearch annotations in tabular format. The process of Pfam vectorization is explained fully and visually in our previous work [25].

Prior to isolate selection, weights are assigned to functions. Functions deemed to be core to the studied group of metagenomes (>50% prevalence across the corresponding metagenomes) are given an additional weight (default: 0.0005). If two groups of metagenomes were used as input, functions are considered differentially enriched within the studied group compared to the second group based on a Fischer exact test of function prevalence. Functions with a *P*-value < .05 are given an additional weight (default: 0.0012). Users can modify the weighted values. To define the most appropriate weightings for a dataset, the ‘MiMiC2-weight-estimation.py’ script evaluates the selection of SynComs with weights ranging from 0 to 1, at increments of 0.00001, for each weighting strategy individually. The SynComs at each increment are compared to the original samples to provide Jaccard distance values, number of matching functions, and number of mismatching functions, allowing the user to identify the optimal weighting strategy for their dataset.

In the main ‘MiMiC2.py’ script, the first step (Fig. 1A and C) involves sample-specific selection of SynComs using an iterative process [25]. The iterative selection involves comparing each genome’s Pfam vector against each metagenome’s Pfam vector. The number of Pfams present within both vectors (‘matches’) provide a score of one each, whereas the number of Pfams present in the genome but absent from the metagenome (‘mismatches’), provide no score. The weighted scores of matching Pfams are then added to the total score to provide the genome score. This is done for all genomes, with the highest scoring genome being selected for inclusion in the SynCom. The Pfams encoded by the selected genome are removed from consideration, with only those Pfams unrepresented being studied. This process is explained visually in Supplementary Fig. S1. The iterative process is continued until 20 strains have been selected to capture the functional potential of each metagenomic sample. The rational for selecting 20 strains is that previous knee point calculations to identify the optimal number of strains required to cover the functionality of metagenomes ranged from 7 to 10, yet this captured only ~80% of Pfams, missing many rarer functions [25]. To enhance the potential inclusion of rarer functions, we doubled the maximum optimal number of strains required within this initial selection.

Step 2 (Fig. 1A) reduces the landscape of considered genomes. The selection prevalence of each strain is determined and a threshold applied (default: 33.3% of predicted sample-specific strain selections). The prevalence threshold should be modified to ensure sufficient strains are shortlisted to provide diversity within the downstream steps. Only strains selected in at least the threshold percentage of samples are kept and further used for the creation of the SynComs. Next, all potential combinations of the shortlisted genomes are generated, considering the user-defined number of members (default: 10). SynCom size should be modified based on the complexity of the ecosystem being studied and strains available. These combinations are then filtered based on the taxonomic assignment of the isolates to enhance diversity within the SynComs and reduce potential competition for carbohydrate utilization between closely related isolates [41, 42]. For example, a SynCom that contains two members of the same species would be filtered out if taxonomic filtering is conducted at the species level. By default, taxonomic filtering is at the species level, but it can be altered to any taxonomic level. The taxonomic assignment file is accepted in the format of GTDB-Tk output [43].

Step 3 (Fig. 1A and D) compares each of the filtered SynCom combinations against the samples of interest. This process is explained visually in Supplementary Fig. S1. To study the combination of strains as a single unit, the Pfam vectors of all SynCom members are combined into a single binary vector that captures the functionality of the entire SynCom. This combined vector is compared against each of the metagenomic samples of interest. The average SynCom score is calculated and termed the ‘Group score’. The SynCom combination with the highest group score is selected.

Step 4 (Fig. 1A) involves simulation of metabolic interaction between members of the best scoring combination of genomes to infer the stability of the community. By conducting initial *in silico* experiments, we aim to reduce the prediction of SynComs that functionally represent a set of metagenomes, but cannot form a cohesive community together. For this, metabolic models for each genome are required [38]. Metabolic models of each member are then simulated over 7 h of growth individually, paired with each other member, and as an entire community. This is done within the BacArena toolkit [39]. Changes in growth within each pairwise interaction was compared to the constituent strains individual growth, with a 10% change in growth deemed large enough to be positive (>10%) or negative (<10%) interaction. Interactions were grouped as mutualism, parasitism, commensalism, amensalism, or neutralism [44]. To be determined as viable, over 50% of the pairwise iterations must be neutral or positive. This is to ensure niche differentiation occurs within the SynCom [45], and was set at 50% to remove only the most competitive ecosystems compared to experimental testing of SynComs, which provide rates of non-negative interactions at ~85% [44]. Secondly, each strain must have at least one positive interaction, confirming mutualistic interactions such as potential cross-feeding, which enhances the stability of a community [46]. Finally, we require that each member must grow within the community model, ensuring a niche can be obtained for each strain rather than strain exclusion. If the SynCom combination with the highest group score (step 3) fails to meet these requirements, the next highest group scoring combination is tested until a stable community is identified. Once identified, a range of graphs and tables are produced detailing the quality of the SynCom for the user.

### Synthetic community stock creation

Cryostocks of SynComs were prepared in faecal microbiota transplantation (FMT) medium in an anoxic workstation (MBraun GmbH, Germany, gas composition 89.3% N_2_, 6% CO_2_, 4.7% H_2_). SynCom strains were grown in monocultures in their preferred growth medium for 24 h. Strain identity was ensured with a MALDI-TOF MS (Bruker Daltonics, Bremen, Germany). Equal volumes from each 24-h growth monoculture were taken, mixed to form the SynCom, and then diluted 1:1 with anoxic FMT medium. The mixture was vortexed before aliquoting (1 ml per tube), freezing on dry ice, and storage at −75°C until usage. Stock purity was ensured by 16S rRNA gene amplicon sequencing.

### Serial batch fermentation in Hungate tubes

A stock solution (1 ml) was inoculated into 9 ml of anoxic brain heart infusion (BHI) medium (Oxoid, CM1135B) in a Hungate culture tube and incubated at 37°C for 24 h (Day 0). The SynComs were then transferred into a new Hungate tube containing fresh medium, again 1 ml culture into 9 ml anoxic BHI (Day 1–5). The experiments were carried out in biological triplicates (i.e. independent cultures started from separate cryoaliquots). Optical density in each Hungate was measured at 600 nm right after transfer to the next tube with fresh medium and after 24 h of growth. After 5 passages, samples were processed for 16S rRNA gene amplicon sequencing and metabolite profiling by high-performance liquid chromatography (HPLC). Therefore 1 ml culture was separated into their pellet and supernatant fraction by centrifugation (10 000 × g, 10 min, 4°C). Samples were stored at −75°C until further usage.

### Metabolite profiling

Concentrations of short-chain fatty acids (acetate, butyrate, propionate, and valerate), branched chain fatty acids (isobutyrate and isovalerate), intermediate metabolites (ethanol, formate, lactate, 1,2-propandiol, 1-propanol, and succinate), as well as mono- and disaccharides (galactose, glucose, and lactose) were acquired as described previously [35]. External standards (HPLC grade compounds; Sigma-Aldrich) were used for concentration determination by comparison of peak retention times. Peaks were integrated using the Chromaster System Manager Software (Version 2.0, Hitachi High-Tech Science Corporation 2013, 2017). Metabolite concentrations >0.2 mM (limit of detection (LOD) for citrate, 1-propanol), >0.24 mM (LOD for butyrate, formate, galactose, glucose, isobutyrate, isovalerate, lactose, and valerate), >0.4 mM (ethanol, 1,2-propandiol), and > 0.8 mM (acetate, lactate, propionate, and succinate) were considered for analysis if present in all three replicates. Production and consumption of metabolites was calculated by subtracting the baseline values from the sample taken after 24 h of growth.

### Batch experiments in human simulated colonic medium with lumen and mucus-microenvironments

Batch experiments were carried out in 50 ml penicillin bottles containing 19 ml of human simulated colonic nutritive medium (adapted from previously published work [47]) and 50 mucin-alginate beads to distinguish lumen from mucus-associated gut microbes. The nutritive medium was flushed under CO_2_ flow and autoclaved before use. Penicillin bottles were inoculated with 1 ml of SynCom and incubated at 37°C for 24 h under agitation (100 rpm). Aliquots were taken immediately after inoculation (T0) and after 24 h fermentation for cell density determination by plating on BHI agar medium (storage at −80°C).

### HuSynCom *in vivo* experiment

For colonization experiments with HuSynCom, germfree male and female wildtype BALB/cJ mice were kept in positive-pressure isolators at the germ-free animal facility of the German Institute for Human Nutrition Potsdam-Rehbruecke (Nuthetal, Germany) with a 12 h light–dark cycle at 22 ± 2°C and 55 ± 5% air humidity. Germ-free mice were colonized by oral gavage at day 0 and 3 with HuSynCom. After 4 weeks, mice were culled and gut luminal content taken for further analysis. These experiments were approved by the Ministry of Social Affairs, Health, Integration and Consumer Protection of the state Brandenburg (permit no. 2346-15-2021).

### IBD-SynCom *in vivo* experiments

Germfree male and female C57BL/6J.129P2-*Il10^tm1Cgn^*/JZtm (IL10^−/−)^ mice were obtained from the Central Animal Facility (Hannover Medical School, Hannover, Germany). Breeding was performed in plastic film isolators (Metall+Plastik GmbH, Radolfzell-Stahringen, Germany) located in a room with a controlled environment (21 ± 1°C, 50%–55% humidity) and 14-h light/10-h dark cycles. Health monitoring of the germfree mouse population was performed according to recommendations for maintaining gnotobiotic colonies and FELASA recommendations [48, 49]. All animals were proved to be free of contaminants or infections. For the experiments, mice were transferred and maintained in airtight cages with positive pressure (IsoCage P, Tecniplast Deutschland GmbH, Bavaria, Germany) to keep their gnotobiotic status. Mice received pelleted 50kGy gamma-irradiated feed (Complete feed for mice – breeding (M-Z), V1124–927; Ssniff Spezialitäten GmbH, Soest, Germany) and autoclaved water *ad libitum*. After weaning, germfree IL10^−/−^ mice were colonized with one of the two bacterial communities (IBD-SynCom or nonIBD-SynCom) by oral and rectal gavage on 2 days (d0 and d3, each 50 μl per route/animal). Faecal samples were collected to screen for the presence of SynCom members (16S rRNA gene amplicon sequencing) and faecal lipocalin-2 levels (ELISA) at the end of the experiment. After 8 weeks of colonization, mice were culled by CO_2_ inhalation followed by exsanguination via cardiopuncture. Tissue samples for relative gene expression analysis (proximal colon) and histopathology (colon, including rectum) were harvested. The experiment was performed twice and each SynCom cohort was maintained in 3–4 different cages consisting of at least two mice per IsoCage. All procedures were approved by the local institutional Animal Care and Research Advisory committee and permitted by the Lower Saxony State Office for Consumer Protection and Food Safety or the local veterinary authorities (reference numbers: 42500/1H and 19/3187).

### Histopathology

The colon was collected and fixed in neutral buffered 4% formalin. Subsequently, samples were dehydrated, embedded in paraffin, sectioned at 3 μm, and stained with hematoxylin and eosin (H&E). The stained colon sections were scored blindly for the proximal, middle, and distal part separately. Parameters for histopathological lesions (ulceration, hyperplasia, severity, and the involved area) were graded from 0 (physiological) to 3 (severe changes) and added in a total score from 0 to 36.

### Relative gene expression analysis

For gene expression analysis, samples of the proximal colon (~0.5 cm) were rinsed with sterile PBS, quickly frozen in liquid nitrogen, and stored at −80°C until further analysis. Total RNA was extracted from the proximal colon tissue using the RNeasy Kit (Qiagen, Hilden, Germany), which included an additional on-column DNase digestion step (RNase-Free DNase Set, Qiagen, Hilden, Germany). cDNA synthesis was performed with the QuantiTect Reverse Transcription Kit (Qiagen, Hilden, Germany) following the manufacturer’s guidelines. The cDNA samples were then normalized to the concentration of 25 ng/μl using HPLC grade water (J. T. Baker, Deventer, Netherlands). Quantitative PCR (qPCR) was conducted using QuantiTect Primer Assays to assess claudin-4 gene expression (Mm_Cldn4_1_SG, Qiagen) or TaqMan Gene Expression Assays for tumour necrosis factor (TNF) level analysis (Mm_00443258_m1, ThermoFisher Scientific), as per the manufacturer’s instructions. Beta-actin served as the reference gene in both assays (Mm_Actb_2_SG and Mm_00607939_s1). Detection was carried out using the QuantStudio 6 Flex Real-Time PCR System (Applied Biosystems, Weiterstadt, Germany) with either the Fast SYBR Green Master Mix or TaqMan Fast Advanced Master Mix, according to the manufacturer’s specifications. All reactions were performed in triplicate. The thermocycling conditions for SYBR Green chemistry included: (i) a polymerase activation step of 20 s at 95°C; and (ii) 40 cycles of 3 s at 95°C and 30 s at 60°C (annealing and elongation step). The amplified PCR product was verified by melting curve analysis (for SYBR Green chemistry). The thermocycling conditions for TaqMan chemistry were: (i) an incubation step of 2 min at 50°C; (ii) a polymerase activation step of 2 min at 95°C; and (iii) 40 cycles of 1 s at 95°C and 20 s at 60°C (annealing and elongation step). Relative gene expression was calculated using the 2^−ΔCt^ method.

### Measurement of faecal lipocalin-2 levels (ELISA)

Frozen faecal samples were homogenized in cold 0.9% NaCl (1 g faeces in 10 ml) to get homogenous faecal suspension. Samples were then centrifuged for 15 min at 15 000 × g and 4°C. Supernatants were transferred into a new vial and further diluted with 0.9% NaCl (1/50 for uninflamed samples and 1/1000 for inflamed samples). Lipocalin 2 levels were detected in the supernatants using Mouse Lipocalin-2/NGAL DuoSet ELISA (R&D Systems, Minneapolis, MN) following manufacturer’s instructions.

### 16S rRNA gene amplicon sequencing

Samples were processed and analysed as described previously [50]. In brief, metagenomic DNA was purified on columns (Macherey-Nagel) after mechanical lysis by bead-beating. The V3-V4 region of the 16S rRNA genes were amplified (25 cycles), then purified with a pipetting robot using AMPure XP magnetic beads (Beckman-Coulter, Germany). Sequencing was conducted in paired-end mode using the v3 chemistry (600 cycles) on a MiSeq System (Illumina) according to the manufacturer’s instructions. Raw sequencing reads were processed using IMNGS [51], which is based on UPARSE [52]. Initial clustering was conducted at 100% sequence identity to create zero space operational taxonomic units (zOTUs). These were then re-clustered at 97% sequence identity to produce species-level operational taxonomic units (OTUs). Only zOTUs and OTUs that occurred at a relative abundance ≥0.25% in at least one sample were kept for further processing [53]. Normalization of samples was conducted in R using Rhea [54]. Sequences were assigned to SynCom members using blastn (E-value <1e−25, 97% identity, 80% query coverage) [55] to compare the amplicon sequence to the 16S rRNA gene sequences of isolates.

### Ecological analysis

To study the ecology of strains within the human gut, 1000 16S rRNA gene amplicon samples labelled as ‘human gut’ from the IMNGS database [51] were obtained. OTU sequences were assigned to SynCom members by comparison against the 16S rRNA gene sequences of SynCom members using blastn (E-value <1e−25, 97% identity, 80% query coverage) [55].

### Phylogenomic analysis

Genomes were taxonomically assigned either based on their published, curated taxonomic assignments, or based on GTDB-Tk v.2.2.1 [43] assignment against the GTDB database v214 [37]. Phylogenomic trees were generated by predicting the protein sequences within each included genome using PROKKA v1.13 [56] as input for Phylophlan v3.1.1 [57], with the ‘—diversity high -f supermatrix_aa.cfg’ options.

## Results

### Comparison of methods for the functional selection of strains

The rationale behind strain selection as part of a SynCom was to maximize the inclusion of functions encoded by a metagenome, without adding functions not detected in the original community. The selection is optimized for SynComs that are representative of an entire group of samples, rather than individual samples, as detailed in the methods (Fig. 1A and C).

**Figure 2:**
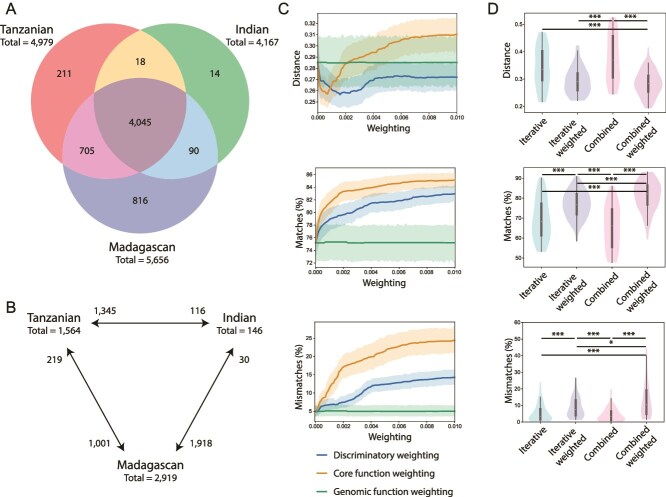
Benchmarking the weighting and selection strategy for SynComs. (A) The overlap in core functions identified within the metagenomes of each population (Tanzanian, Indian, Madagascan) (*n* = 20 metagenomes for each population). (B) The number of differentially present functions identified by pairwise comparison of the populations. The total number of functions weighted during SynCom selection for each population is stated below the populations name. (C) The three weighting strategies were evaluated individually, studying the impact of increasing the weight used on the separation of functional profiles from three distinct human populations, represented by their associated colour on the three plots. The impact was determined based on their Jaccard distance, percentage of matching Pfams, and the percentage of mismatching Pfams, when compared to their original samples. Each weighting was scored from 0 to 0.01, in steps of 0.0001, creating a continuum of scoring. (D) Comparison of iterative or combined scoring on SynCom selection. In tandem to the selection methods (iterative or combined), the impact of weighting functions was assessed. For each combination, three SynComs were created, one representative of each population, which were compared to their respective samples for a total of 60 comparisons. Statistical significance is represented as: ^*^ < .05, ^**^ < .01, ^***^ < .001.

With the aim to select a single SynCom that functionally represents a group, we investigated whether applying additional weight to specific functions improves group representation. The three weighting strategies were: core metagenomic function, individual genome function, and discriminatory weighting. Core function weighting enhances the selection of functions that are core to a group of metagenomes (i.e. present in most samples). Genomic function weighting increases the selection of functions rarely encoded by strains in a collection. Discriminatory weighting identifies functions differentially present in two groups of samples (e.g. optimal versus disturbed communities) allowing functions enriched within a group of interest to be weighted and hence preferentially selected. The impact of these functional weighting strategies on SynCom selection was tested using a collection of 60 human gut metagenomes, 20 each from 3 different populations (Tanzanian (Hadza tribe), Indian, Madagascan) (Supplementary Table S1). Core functions were identified in each of the populations and compared, showing a conserved collection of 4045 functions that were core across the 3 populations (Fig. 2A). Discriminatory functions were identified by pairwise comparison of the three populations samples to each other (Fig. 2B). Isolates (*n* = 219) from the human intestinal bacteria collection (HiBC) [35] were used as input, as they originate from the same ecosystem. We compared the combined Pfam vector of the selected SynComs against the Pfam vector of the original sample they represent using: Jaccard distance, percentage of matching Pfams, and percentage of mismatching Pfams.

The impact of each three weighting strategies was tested separately using weights from 0 to 1, in increments of 0.0025, across the 60 metagenomic samples for a total of 72 000 data points (Supplementary Table S2). We found that weightings for all strategies above 0.1 had no noticeable effect on the three metrics, with positive effects occurring with weights below 0.01 (Supplementary Fig. S2). Therefore, a second set of analyses was conducted using weights from 0 to 0.01, in increments of 0.001 (*n* = 100), across the 60 samples for a total of 6000 data points (Fig. 2C, Supplementary Table S3). Although both core metagenomic function and discriminatory weighting had a significant effect on all metrics examined, genomic function weighting had no effect and was not explored further. Smaller weights (<0.004) decreased distance compared to no weighting, whereas larger weights increased distance to the original samples. The percentage of matches and mismatches was increased by the addition of any weight in both strategies (core metagenomic function and discriminatory weighting). A discriminative weight of 0.0012 significantly decreased the Jaccard distance to the original samples (*P* < .001) from 0.29 ± 0.09 to 0.28 ± 0.08 and also significantly increasing the number of functional matches (*P* < .001) from 75.21 ± 11.28 to 76.31 ± 10.73. The addition of a core metagenomic function weighting score of 0.0005 also significantly decreased the distance to the original samples (*P* < .001) from 0.29 ± 0.09 to 0.26 ± 0.05 and also significantly increasing the number of functional matches (*P* < .001) from 75.21 ± 11.28 to 80.31 ± 7.28. Based on these results, the default weightings used throughout this paper are 0.0012 for discriminative weighting and 0.0005 for core function weighting.

In parallel to weighting, we determined the effect of selecting members of a SynCom iteratively or together as a combined entity (Supplementary Fig. S1A; Fig. 2D), which is included in ‘Step 3’ of the workflow (Fig. 1C). The iterative selection of strains selects the best scoring strain in each round, but then ignores all functions encoded by previously selected strains. Alternatively, the combined process creates a single Pfam vector for each potential SynCom consisting of the filtered list of strains from ‘Step 2’, and focuses on selecting the strains together, rather than one at a time. The combined process had the largest distance to the original samples, however, in tandem with the weighting strategies, it resulted in the smallest distance. The tandem of combined selection and weighting also had a significantly higher number of matches compared to the application of iterative selection and weighting (*P* < .001). Weighting increased the number of mismatching functions within a SynCom, although this had no noticeable effect on the distance of the SynComs to their original samples (Supplementary Fig. S1B–E). In summary, the application of core and discriminatory weighting are beneficial to the selection of SynComs functionally representative of native microbiota samples.

### Representative synthetic communities of gut microbiomes

**Figure 3:**
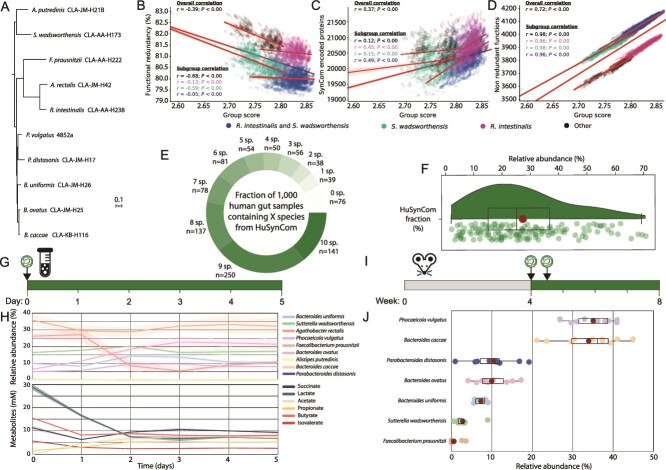
Development and testing of a SynCom of the human gut. (A) Phylogenomic tree of HuSynCom along with strain identifiers. (B–D) All three plots are based on the 13 662 SynComs studied during selection, with the functional redundancy, number of proteins encoded by a SynCom, or number of nonredundant functions encoded compared against the SynComs group score. Each SynCom is represented by a point, coloured based on the inclusion of *R. intestinalis*, *S. wadsworthensis*, both of these species, or neither within the SynCom. The correlation values are coloured depending on the group they represent, with a separated set of values representing the overall correlation. (E) Prevalence of the HuSynCom species within 1000 human gut-derived 16S rRNA gene amplicon datasets. (F) Relative abundance of the microbiota accounted by HuSynCom in samples containing all 10 species (*n* = 141). (G) Experimental design of serial batch fermentation in Hungate tubes over 5 days. (H) Community dynamics studied via 16S rRNA gene amplicon sequencing and metabolite profiles using HPLC-RI of the serial batch fermentation over 5 days. Samples were taken once per day, during passaging of the community. (I) Experimental design of colonization of germ-free wildtype BALB/cJ mice with HuSynCom. (J) Relative abundances of the bacterial strains in the colon of the gnotobiotic mice four weeks after colonization. Each dot represents an individual mouse; 8 dots are present for *F. prausnitzii* as it was not detected in the colon of two mice. HuSynCom species are coloured consistently across figures.

The mammalian gut is one ecosystem that varies greatly between hosts and significantly impacts their hosts health and development. The importance of the gut microbiota to human health has led to the creation of several SynComs to facilitate targeted studies of host–microbe interactions (e.g. SIHUMI [9], SIM [58], MCC100 [59], and hCom2 [10]), yet only SIHUMI consists of publicly available strains, allowing it to be used by the research community. Given the importance of this ecosystem, and availability of publicly accessible strains [35], we developed a functionally selected SynCom representative of the human gut microbiota. For this, we used a cohort of 179 healthy American metagenomes to select a SynCom of 10 members [26] together with publicly available isolates that can be used globally [35]. Of the 219 human gut strains, 178 were selected during the iterative generation of sample-specific SynComs, and 18 were kept due to their high prevalence. With a SynCom size of 10, these 18 strains combined to produce 43 758 potential SynComs, which was reduced to 13 662 after omitting SynComs containing multiple strains of the same species. Comparison of the functions encoded by each SynComs to the healthy American cohort identified that the highest-scoring SynCom accounted for an equal number of functions within the input metagenomes as the sample-specific SynComs of the same size (*P* = .59). We termed this SynCom representing the human gut microbiota ‘HuSynCom’, which contains representatives of 8 genera, including three *Bacteroides* spp. (Fig. 3A).

Using the 13 662 combinations of strains considered during selection of HuSynCom, we investigated which features determined SynCom selection. Due to the restricted number of strains included within SynComs (*n* < 20), functionally complementary strains that did not encode for the same protein families are preferentially selected, hence should have a low overlap in their functionality, termed the functional redundancy. Functional redundancy was calculated as the percentage of unique Pfams encoded by a SynCom compared to all Pfams encoded by the SynCom. This was confirmed by the significant overall negative correlation of group score with functional redundancy encoded by the SynComs (Fig. 3B). This suggests that members of the highest scoring SynComs encode unique functions, enhancing the ability of SynComs to occupy different niches. Although each HuSynCom member encoded unique functions, >50% of functions were encoded by four or more members, highlighting extensive functional redundancy (Supplementary Table S4). The group score weakly correlated with the total number of proteins encoded by a SynCom (Fig. 3C), but strongly correlated with the number of non-redundant proteins (Fig. 3D). This suggests that SynComs that encode a greater number of functions (nonredundant Pfams) are more able to functionally represent the functional complexity of a microbiota. Correlation of nonredundant functions with the group score identified two parallel groups of SynComs with the same trend, separated by ~200 Pfams (Fig. 3D), caused by the presence of *Sutterella wadsworthensis* within a SynCom. *S. wadsworthensis* encoded a unique combination of 203 Pfams not encoded by any other strains. A secondary pattern within the data was also observed, with SynComs with the highest group scores including *Roseburia intestinalis*, which encoded 167 Pfams not present in any other strains. These 167 Pfams were also highly prevalent in the target metagenomes, which encoded an average of 67.9% (113 ± 34) of these functions. Given the patterns described, SynComs that included both, *S. wadsworthensis* and *R. intestinalis* had the highest group score and encoded the largest functional repertoire.

HuSynCom contains many common members of the human gut; if they form a stable community within native human gut samples, we expect them to co-occur. Across 1000 human gut 16S rRNA gene amplicon samples, >50% (*n* = 528) contained 8 of the 10 HuSynCom species (Fig. 3E). This confirmed that most of these species commonly co-occur within the human gut microbiota. In addition, HuSynCom members represented a dominant fraction of sequencing diversity, covering a cumulative relative abundance of 27.3 ± 16.4% in samples containing all 10 members (Fig. 3F).

Metabolic modelling supported that HuSynCom forms a stable community, with minimal changes in bacterial composition over 7 h (Supplementary Fig. S3). We then performed *in vitro* experiments to validate this *in silico* finding. Serial batch fermentation in BHI medium over 5 days confirmed the stability of the community (Fig. 3G), both taxonomically and functionally, with 9 of the 10 bacteria detected by amplicon sequencing (Fig. 3H). During the first 2 days, the community fluctuated but reached an equilibrium that was maintained for the following 3 days. The ability to utilize mucin is a feature of many gut microbes, leading them to colonize the mucin layer of the gut. To identify if HuSynCom captured this biogreographical variability, we modelled the community in the Virtual Colon system [40] and identified that *S. wadsworthensis* preferentially colonized the mucus layer (Supplementary Fig. S4A). Although *Agathobacter rectalis* (synonym *Eubacterium rectale*) also colonized the mucus layer, it was not predicted to utilize N-acetylneuraminate, a common glycan in mucus, whereas *Phocaeicola vulgatus* did (Supplementary Fig. S4B). Batch fermentation in simulated human colonic medium with mucin-alginate beads to simulate the mucus-associated microbiota confirmed both *P. vulgatus* and *S. wadsworthensis* dominated the mucin microenvironment (Supplementary Fig. S5). We next performed gnotobiotic mouse experiments to assess the ability of HuSynCom to colonize the intestine (Fig. 3I). After 4 weeks, 7 of the 10 strains were detected by amplicon sequencing, resulting in a community dominated by *Bacteroides caccae* and *P. vulgatus* (Fig. 3J). *Faecalibacterium prausnitzii* colonized the mice sporadically, being detected in 6 of 10 colon samples, whilst it dominated the community in the serial batch cultures.

To highlight the potential of the SynCom design approach to functionally select defined communities for other ecosystems, we created SynComs for other mammalian gastrointestinal ecosystems. The gastrointestinal microbiota of ruminants directly influences both host growth [60] and contributes to global methane emissions [19], however, no SynComs exist for this ecosystem. Using metagenomes from 78 bovine rumen samples [60] and the Hungate1000 genome collection [36] (*n* = 410), we created a rumen SynCom called ‘RuSynCom’ (Supplementary Fig. S6). In contrast to the rumen, there are several SynComs representing the mouse gut against which we can compare our selection, including the well-studied OMM12 model SynCom [6, 61, 62]. We analysed 500 mouse gut metagenomes to select a SynCom consisting of 12 members from the miBC2 isolate collection [7, 63], called ‘MuSynCom’ (Supplementary Fig. S7). Metabolic modelling of the two SynComs confirmed OMM12 is dominated by ammensalistic interactions (33.33%) [44], whereas MuSynCom interactions were mostly commensal (45.45%) (Supplementary Fig. S7B and C). To facilitate the creation of SynComs by others, we have generated the required files for publicly available isolate collections from the pig [34], human [35], mouse [7], and ruminant [36] gastrointestinal tracts. To further facilitate the design of SynComs for any ecosystem, we have also vectorized all genomes within the GTDB (r202) [43]. This information can guide researchers as to which taxa are required to represent an ecosystem, facilitating targeted isolation and characterization.

### Recapitulation of host phenotypes using disease-specific synthetic communities

**Figure 4:**
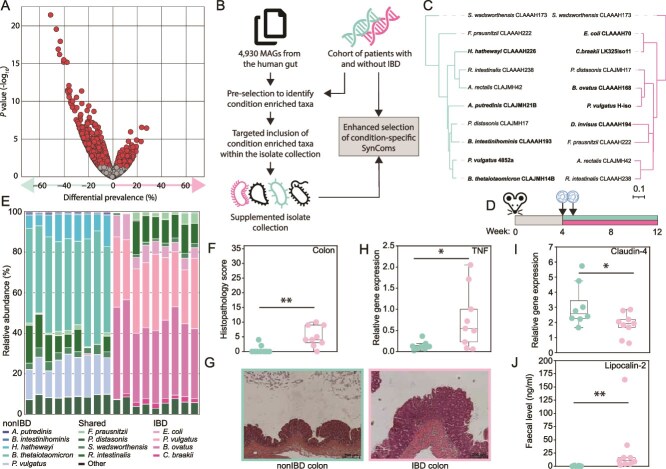
Creation of model SynComs to study chronic intestinal inflammation. (A) Differential prevalence of Pfams between the IBD and nonIBD samples. (B) MAG-based workflow to enrich isolate collections prior to SynCom selection. (C) Phylogenomic tree of each SynCom, including strain identifiers. (D) Design of the germ-free experiments with the IBD- and nonIBD-SynComs. (E) Colonization of SynCom strains in the colon 8 weeks after gavage (16S rRNA gene amplicon sequencing). (F) Histopathology scoring of colonic tissue. (G) Representative images of the colon (transversal sections). (H) TNF gene expression within the proximal colon as quantified by qPCR. (I) Claudin-4 gene expression within the proximal colon as quantified by qPCR. (J) Faecal lipocalin-2 at 12 weeks of age as quantified by ELISA.

A major benefit of SynComs is that they reduce the inherent variation due to both, the complexity and large unknown fraction in complex microbial communities. One of the most studied examples of microbiota-driven human disease are inflammatory bowel diseases (IBD). Although common features of IBD-associated microbiota have been identified [26, 64, 65], no model community for the study of these diseases exist. Therefore, we used metagenomic data from a previously published cohort of patients with ulcerative colitis (UC), a subtype of IBD (*n* = 167), and individuals without inflammation (*n* = 178) to select SynComs for both conditions [26]. To facilitate the creation of communities based on the comparison of two groups of samples, we compared function prevalence between the groups, identifying 1361 functions enriched in nonIBD and 401 enriched in the UC samples (Supplementary Table S5, Fig. 4A). To enhance the inclusion of these differential functions within the SynComs, they were weighted as described above (Fig. 2).

Prior to generating SynComs, we wanted to confirm our isolate collection included all taxa required to capture the functionality associated with IBD. Therefore, a comprehensive set of MAGs from the human gut [2] was used that included all species observed within the gut, and hence allowed their potential inclusion (Fig. 4B). Of the 4930 MAGs analysed, 873 were selected to represent at least one sample during the iterative process (Supplementary Table S6). The inclusion of *Pseudomonadota*, previously associated with colitis [66, 67], was higher in inflammation-associated MAG-SynComs (34.66% of selected strains compared to 3.09% in controls). This led to the inflammatory representative MAG-SynCom containing five *Pseudomonadota* species, versus none in the control MAG-SynCom (Supplementary Fig. S8). Based on these *in silico* predictions of the taxa most representative of the IBD and nonIBD samples, we chose to enrich our isolate collection with additional *Pseudomonadota* prior to SynCom selection. An additional 11 human gut *Pseudomonadota* isolates were obtained, representing four additional species of *Citrobacter* and *Klebsiella* (Supplementary Table S7) [35].

Using the amended isolate collection, SynComs for both the IBD and nonIBD microbiota were predicted (Fig. 4C, Supplementary Table S8). Five members of both SynComs were the same, likely covering core functions of the human gut, whereas the other five isolates of each SynCom captured differentially prevalent functions. This included two different strains of *P. vulgatus* being selected for each SynCom, likely due to disease-specific, strain-level diversity within this species [65]. Both communities encoded unique functions, the IBD-SynCom encoded 1110 unique functions, giving it a larger functional landscape compared to the control community. To identify strains that contributed to health-associated functions, their occurrence was studied across 439 healthy control samples from 7 studies (Supplementary Fig. S9A) [26, 68–73]. This identified *Hungatella hathewayi* (strain CLA-AA-H226), a member of the nonIBD-SynCom, as the strain best able to capture the functionality of these samples, containing 499 health-enriched functions (Supplementary Fig. S9B). Although other species contained as many health-enriched functions, the combination of enriched functions encoded by *H. hathewayi* CLA-AA-H226 enhanced its selection to represent healthy microbiota (Supplementary Fig. S9C).

The ability of the SynComs to recapitulate host–microbe interactions was determined by colonizing germ-free IL10^−/−^ mice, an established model of experimental IBD [66, 74, 75], for 8 weeks (Fig. 4D). Each of the SynComs colonized the mice, with nine and eight strains detected in mice given the nonIBD- and IBD-SynComs, respectively (Fig. 4E). *A. rectalis* did not colonize as part of either SynCom and *Dialister invisus* did not colonize within the IBD-SynCom. Both communities had a high relative abundance of *P. vulgatus* (*n* = 18) (24.9 ± 7.7%), although different strains were selected for each SynCom, *Bacteroides* spp. were also dominant, with *Bacteroides thetaiotaomicron* (46.6 ± 5.0%) and *B. ovatus* (38.8 ± 5.0%) in the nonIBD- and IBD-SynComs, respectively. Significantly greater inflammation characterized by infiltration of inflammatory cells and mild hyperplasia of the crypt epithelium was observed in the colon of IL10^−/−^ mice colonized with the IBD-SynCom, whereas little evidence of immune cell activation was found in the colonic tissue of nonIBD-SynCom colonized mice (Fig. 4F and G). This was corroborated by increased expression of TNF in colonic tissue (Fig. 4H). Impaired epithelial barrier function, a feature of active colitis [76], was quantified in the IBD-SynCom mice by decreased expression of claudin-4 (Fig. 4I). Faecal levels of lipocalin-2, a biomarker of intestinal inflammation, were significantly higher in the IBD-SynCom group (*P* = .0002) after 8 weeks of colonization (Fig. 4J), and were already higher after 4 weeks (*P* = .0002) (Supplementary Table S9). The results confirm the SynComs are suitable models to mechanistically study the microbial contribution to intestinal inflammation. We have ensured that both SynComs can be used by other researchers as all strains are publicly available from the German Collection of Microorganisms and Cell Cultures (DSMZ) (Supplementary Table S8) [35].

## Discussion

Understanding microbiomes requires model systems that allow specific modulation with repeatable results. For this reason, SynComs are critical to provide a defined background, but an unbiased rationale for strains selection within SynComs remains elusive. Here, we propose that functional congruence can guide this selection process and created a workflow to generate functionally representative SynComs by applying a group-based selection process. Integrating weights on functions based on differential prevalence between groups also improved the selection of functionally representative SynComs. The correct weighting used for each study will vary, the weighting used within this work was benchmarked using human gut metagenomes and hence the same weights may not be applicable for other ecosystems. Similarly, different prevalence thresholds may be required depending on the number of genomes/metagenomes being analysed (Supplementary Table S10).

The findings suggest functional selection of complementary members for a SynCom reduces metabolic competition between SynCom members. This is supported by previous findings that the reliance of strains in a microbiome on other members, such as via amino acid auxotrophies, has been linked to higher diversity, and enhanced stability [77]. However, studies using data from multiple ecosystems have found negative interactions dominate, and could be critical for community assembly and stability [78]. This proposal is supported by the high level of negative interactions observed in established SynComs, such as OMM12 [44]. Given that microbial interactions involve metabolic competition [77], bacteriocin production [24], and altered pH [79], it is likely that a mixture of positive and negative interactions are required for optimal stability [80]. Our approach of quantifying functional redundancy as the encoding of redundant Pfams also fails to account for alternative pathways that achieve the same metabolic process. As many of the Pfams remain functionally unassigned, further study is required to access this in greater depth.

Application to the human gut confirmed that the selected communities functionally represent the ecosystem and captures dominant microbes. In investigating the rationale for strain selection, we observed that strains encoding unique functionality within a collection are more likely to be selected due to a lack of potential redundancy. This enhances the selection of low abundance microbes that encode critical functionality, as shown by the selection of a methane producer (*Methanobrevibacter millerae*) as part of a bovine rumen SynCom, although they account for <0.1% of the microbiota [19, 81]. Selection of such strains is dependent on isolates being available, hence larger publicly available isolate collections are needed as input [43]. Alternatively, MAG collections can be used to identify critical taxonomic groups, allowing targeted isolation and expansion of a culture collection [82]. Using this method to generate disease-specific SynComs allowed the inflammatory phenotype of chronic colitis microbiota to be captured. In addition to creating general disease-specific SynComs, this approach can generate sample specific SynComs, allowing the potential capture of sample-specific microbes that may be rare. Such rare, or inconsistent, microbes are less likely to be selected during the group-wise selection as their absence in some sample would lower the mean score of their associated functions during ‘Step 3’ of the workflow. Additionally, the absence of the microbes from many samples will lower their chance to pass the prevalence filter in ‘Step 2’, prevent their consideration as a member of the group-wise SynCom. For diverse communities where composition is variable, users can stratify their samples based on the composition or functionality, and use these as input, allowing for the creation of SynComs representative of different community states [83]. In addition to creating SynComs based on the functional potential of microbiota via the metagenomes, metatranscriptomes would capture the expressed functionality [84], and enhance sample specific SynCom selection from diverse ecosystems.

Using metabolic modelling of SynComs to identify potential competition between members, we can improve the cohesiveness of output SynComs. However, comparison of predicted results with experimental validation proved difficult, likely due in part to the difference in time scales studied (hours versus days). Although HuSynCom was predicted to be stable, when tested using a variety of *in vitro* and *in vivo* methods, we obtained different colonization patterns. None of the methods allowed the growth of all 10 SynCom members, nine were observed across the studies. *F. prausnitzii* dominated the community during batch fermentation in BHI, but inconsistently colonized germ-free mice. The inability of *F. prausnitzii* strains to colonize germ-free mice, or colonize them at low relative abundance (<0.1%), has previously been reported [85, 86]. These discrepancies may be due to host-specific factors [87] that need quantifying to allow direct comparability between *in vitro* and *in vivo* models. Although metabolic modelling provided initial insights, alternative modelling approaches may provide greater insights into microbe-microbe, and microbe-host interactions [88, 89].

To date, no disease-specific SynComs have been designed to capture the host–microbe interactions in IBD. The approach described in this work was able to distil the functionality of disease-associated microbiota into SynComs representative of either the disease or non-inflamed state. Five strains were consistent between the two SynComs, meaning the remaining five and their interactions with the other community members were the source of the gut inflammatory phenotype. Different strains for *P. vulgatus* were selected for each SynCom, likely due to the large pan-genome of this species allowing both strains to be functionally distinct [90]. Given this species prior functional association with UC, the strains may differ in functions that exacerbate intestinal inflammation [65]. As these SynComs were generated using publicly deposited strains, they can be used as model SynComs for microbiota-induced chronic intestinal inflammation. Future genetic modification of these strains may allow the specific functions involved in the induction of experimental chronic intestinal inflammation to be identified and studied.

## Supplementary Material

Supplementary_Information_wraf209

## Data Availability

The 16S rRNA gene amplicon data have been deposited at NCBI as PRJNA1248391, PRJNA1248442, PRJNA1248459, PRJNA1248715 and are publicly available as of the date of publication. All original code has been deposited at GitHub and is publicly available at https://github.com/thh32/MiMiC2
