# Correction to: Function-based selection of synthetic communities enables mechanistic microbiome studies

**DOI:** 10.1093/ismejo/wrag015

**Published:** 2026-02-21

This is a correction to: Thomas C A Hitch, Johanna Bosch, Silvia Bolsega, Charlotte Deschamps, Lucie Etienne-Mesmin, Nicole Treichel, Stephanie Blanquet-Diot, Soeren Ocvirk, Marijana Basic, Thomas Clavel, Function-based selection of synthetic communities enables mechanistic microbiome studies, *The ISME Journal*, Volume 19, Issue 1, January 2025, https://doi.org/10.1093/ismejo/wraf209

In the originally published version of the paper, the Supplementary Data files were erroneously omitted. The publisher apologises for this error. The files are now supplied to the paper.

