# Supplementary material for: Function-based selection of synthetic communities enables mechanistic microbiome studies
Source: ISME J. 2025 Sep 17;19(1):wraf209. doi: 10.1093/ismejo/wraf209 (PMC12507024; doi:10.1093/ismejo/wraf209)
Supplement: Supplementary_Information_wraf209 [file supplementary_information_wraf209.zip › Figure S2.pdf]

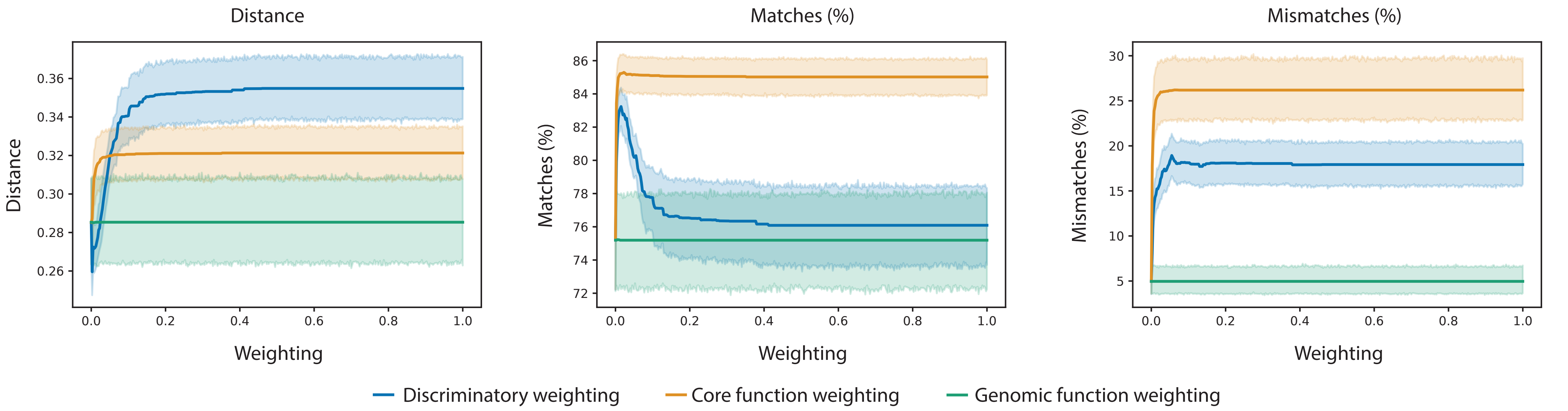

Figure S2: Impact of different weighting strategies on SynCom selection. The functional profile of three distinct human populations (Tanzanian, Indian, Madagascan) were used in this assessment, with three metrics used to determine the impact of weighting strategies on SynCom selection. Each weighting was scored from 0-1, in steps of 0.0025.
