# Supplementary material for: Function-based selection of synthetic communities enables mechanistic microbiome studies
Source: ISME J. 2025 Sep 17;19(1):wraf209. doi: 10.1093/ismejo/wraf209 (PMC12507024; doi:10.1093/ismejo/wraf209)
Supplement: Supplementary_Information_wraf209 [file supplementary_information_wraf209.zip › Figure S3.pdf]

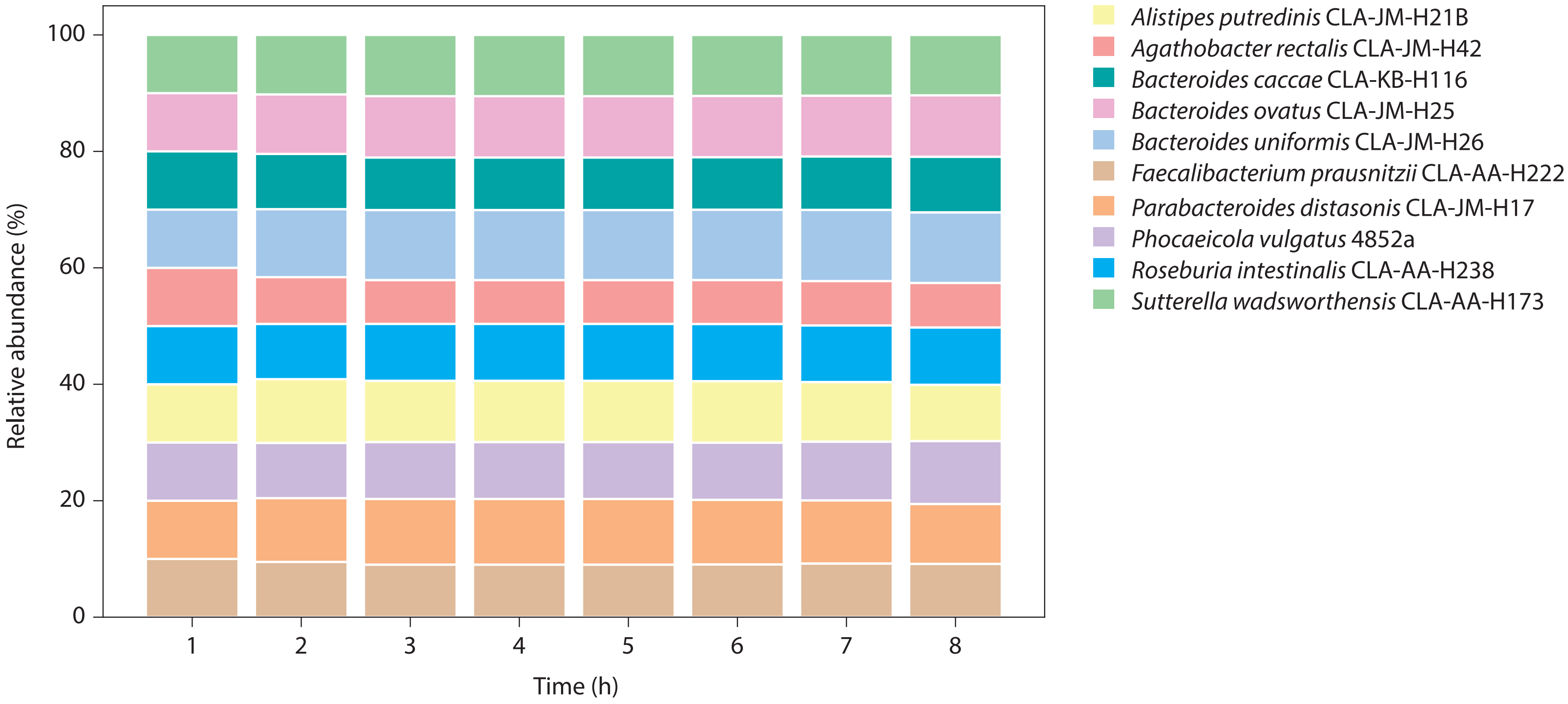

Figure S3: Metabolic modelling of HuSynCom. The relative abundance of each strains contribution to the community was calculated as a percentage from the number of cells from a given strain, divided by the total number of cells.
