# Supplementary material for: Function-based selection of synthetic communities enables mechanistic microbiome studies
Source: ISME J. 2025 Sep 17;19(1):wraf209. doi: 10.1093/ismejo/wraf209 (PMC12507024; doi:10.1093/ismejo/wraf209)
Supplement: Supplementary_Information_wraf209 [file supplementary_information_wraf209.zip › Figure S5.pdf]

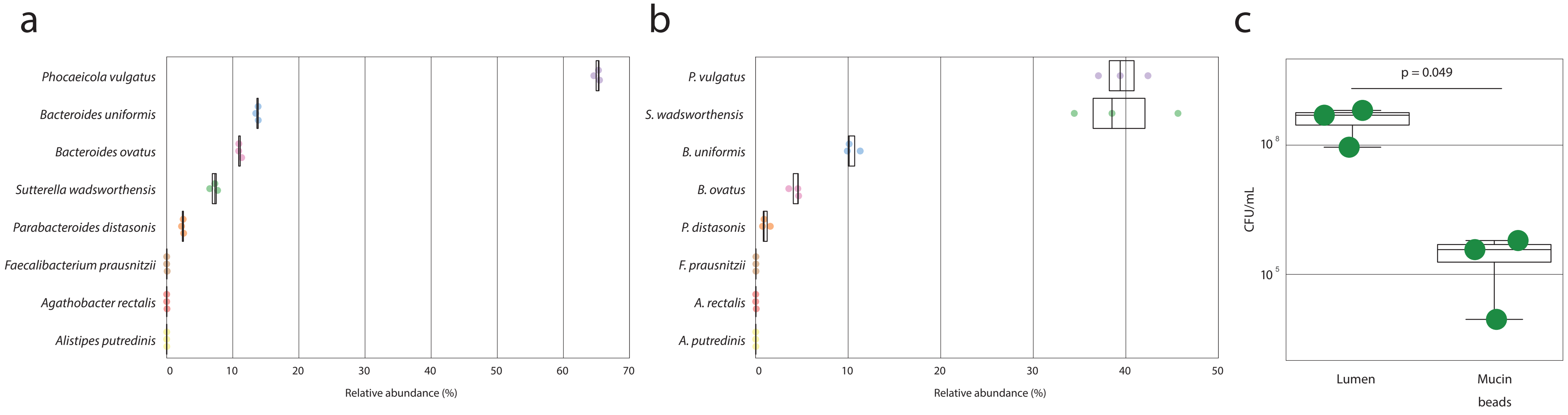

Figure S5: HuSynCom colonisation of a batch fermenter system. a. The taxonomic profile of the luminal content of the batch fermentation. **b**. The taxonomic profile of the mucin bead attached microbiota. Only OTUs present at >0.01% in >=60% of samples were studied. **c**. The colony forming units (CFU) per mL of sample was determined for the luminal content and from the mucin bead associated microbiota. Statistical testing conducted with Wilcoxon rank sum.
