# Supplementary material for: Function-based selection of synthetic communities enables mechanistic microbiome studies
Source: ISME J. 2025 Sep 17;19(1):wraf209. doi: 10.1093/ismejo/wraf209 (PMC12507024; doi:10.1093/ismejo/wraf209)
Supplement: Supplementary_Information_wraf209 [file supplementary_information_wraf209.zip › Figure S6.pdf]

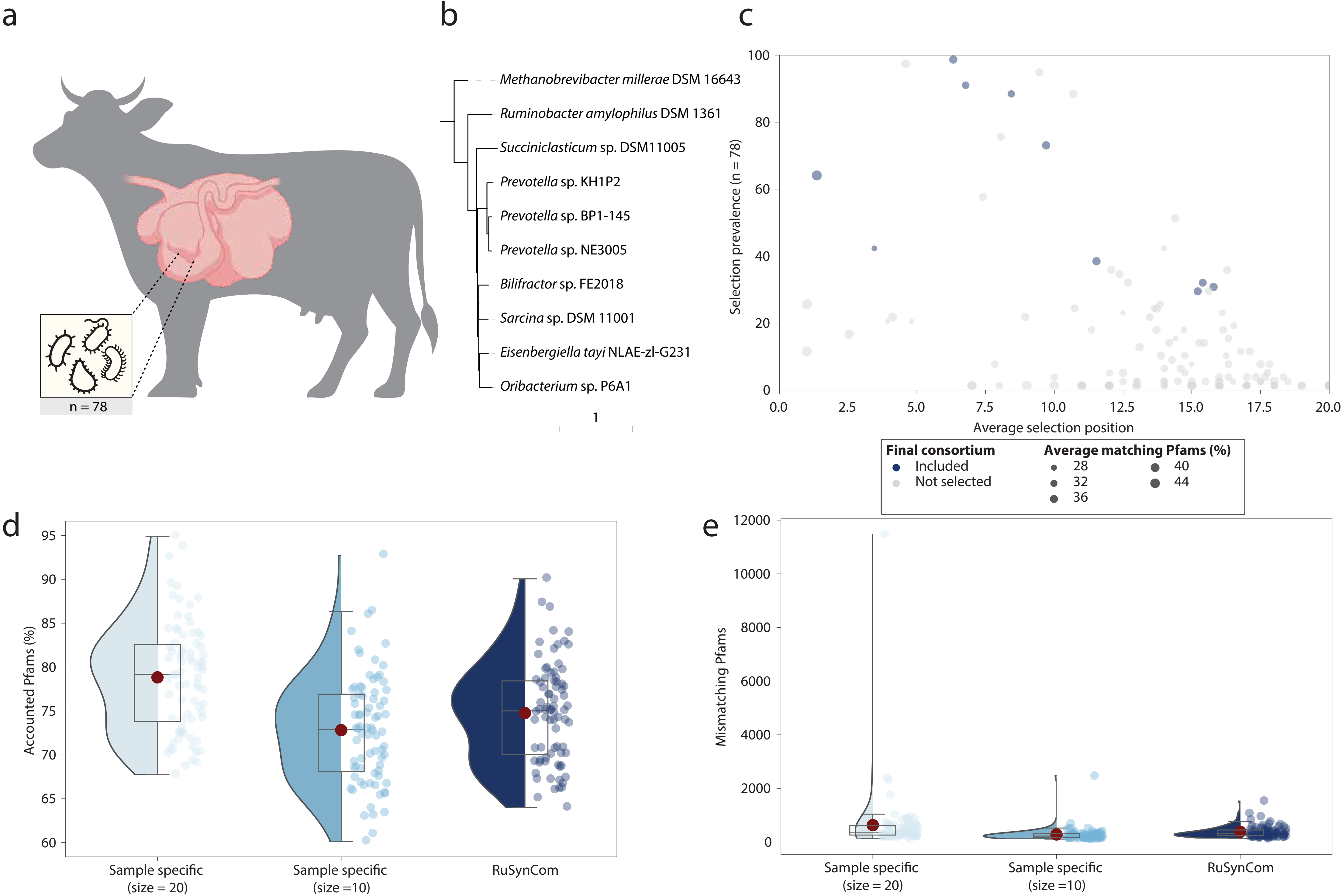

Figure S6: Selection of the rumen SynCom, RuSynCom. a. Selection was based on 78 metagenomes from the rumen of cows. **b.** Phylogenomic tree of RuSynCom members with strain identifiers. **c.** Selection prevalence across the 78 samples and the Pfams captured by each member. **d.** Pfams accounted for by each round of SynCom selection. **e.** The number of mismatching Pfams encoded by each rounds selected SynComs.
