# Supplementary material for: Function-based selection of synthetic communities enables mechanistic microbiome studies
Source: ISME J. 2025 Sep 17;19(1):wraf209. doi: 10.1093/ismejo/wraf209 (PMC12507024; doi:10.1093/ismejo/wraf209)
Supplement: Supplementary_Information_wraf209 [file supplementary_information_wraf209.zip › Figure S7.pdf]

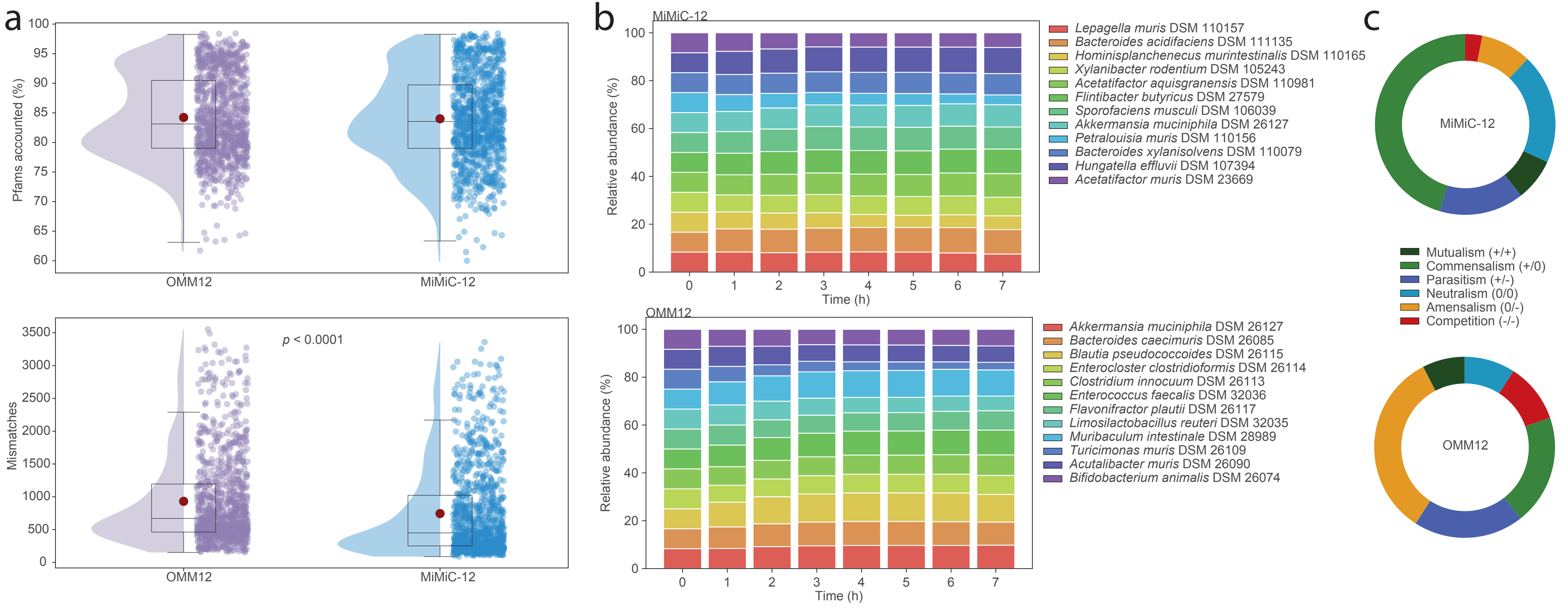

Figure S7: Comparison of SynComs for the mouse gut. a. The Pfams accounted for, and mismatches between both SynComs and1,000 metagenomes from the mouse gut. **b.** Metabolic modelling of the two communities over seven hours. **c.** Pairwise interactions between the members of both SynComs were grouped into pre-defined categories and the frequency of these interactions plotted.
