# Supplementary material for: Function-based selection of synthetic communities enables mechanistic microbiome studies
Source: ISME J. 2025 Sep 17;19(1):wraf209. doi: 10.1093/ismejo/wraf209 (PMC12507024; doi:10.1093/ismejo/wraf209)
Supplement: Supplementary_Information_wraf209 [file supplementary_information_wraf209.zip › Figure S8.pdf]

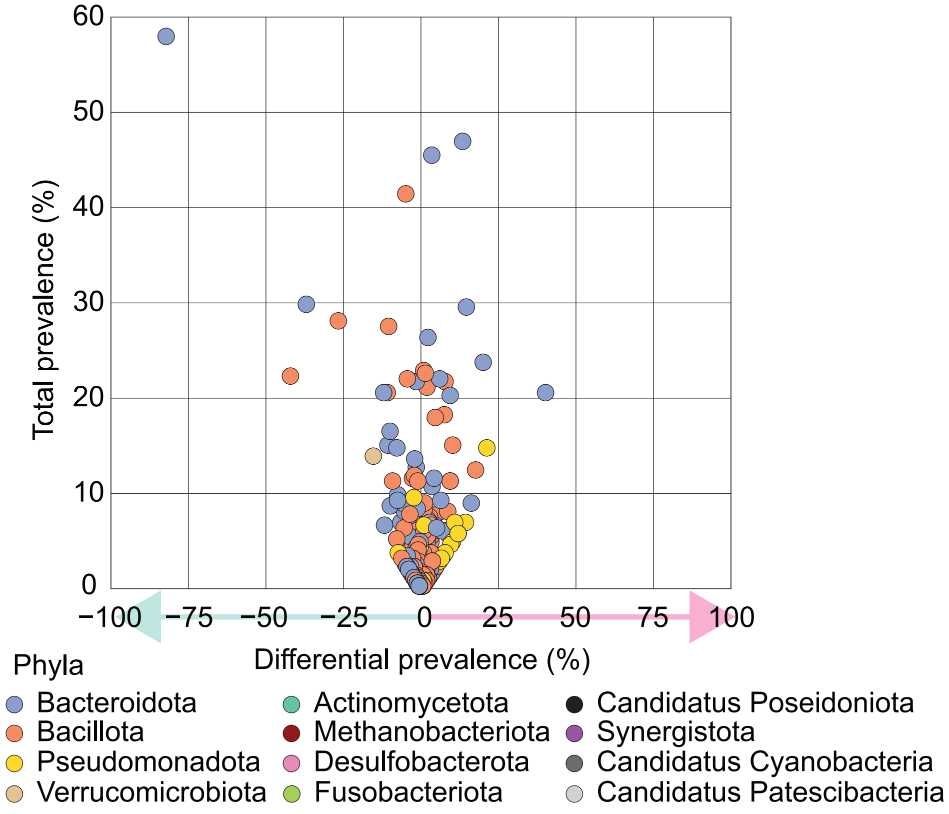


**Figure S8: Initial selection of IBD and nonIBD SynComs based on a MAG collection.** Each dot on the volcano plot represents a MAG which was selected to be part of at least one samples initial SynCom selection. Dots are coloured based on the phyla they belong to.
