# Supplementary material for: Function-based selection of synthetic communities enables mechanistic microbiome studies
Source: ISME J. 2025 Sep 17;19(1):wraf209. doi: 10.1093/ismejo/wraf209 (PMC12507024; doi:10.1093/ismejo/wraf209)
Supplement: Supplementary_Information_wraf209 [file supplementary_information_wraf209.zip › Supplementary Information.pdf]

Supplementary Information

Figure S1: Optimised selection of a single SynCom to represent a group of samples. **a.** Schematic of the iterative process and its application to groups of metagenomes to select a single SynCom. **b-e.** Each plot shows an MDS plot of the functional profiles for each human populations' samples. In addition to the native samples, the functional profile of the SynCom created to represent each population has been plotted as triangles coloured to match the samples they were predicted from. The four SynCom creation methods are the iterative approach (**b**), the iterative approach using the weighting of Pfams (**c**), the combined approach (**d**), and the combined approach using the weighting Pfams (**e**).

Figure S2: Impact of different weighting strategies on SynCom selection. The functional profile of three distinct human populations (Tanzanian, Indian, Madagascan) were used in this assessment, with three metrics used to determine the impact of weighting strategies on SynCom selection. Each weighting was scored from 0-1, in steps of 0.0025.

Figure S3: Metabolic modelling of HuSynCom. The relative abundance of each strains contribution to the community was calculated as a percentage from the number of cells from a given strain, divided by the total number of cells.

Figure S4: HuSynCom colonisation in the VirtualColon simulation. a. Colonisation of the HuSynCom members at 1, 4, and 7 hours of the simulation. **b.** Visualising only the N-acetylneuraminate concentration uncovers the populations within the inner and outer mucus that degraded the mucus layer, and those that utilised alternative sources. The VirtualColon simulation creates three layers of mucus, the lumen (light grey), outer mucus (mild grey) and inner mucus (dark grey). Each are coloured based on their concentration of N-acetylneuraminate.

Figure S5: HuSynCom colonisation in a batch fermenter system. a. Taxonomic profile of the luminal content in the batch fermentation. **b.** Taxonomic profile of the microbiota attached to mucin beads. Only OTUs present at >0.01% in ≥60% of samples were studied. **c.** The colony forming units (CFU) per mL of sample was determined for the luminal content and from the mucin bead-associated microbiota. Statistical testing conducted with Wilcoxon rank sum.

Figure S6: Selection of the rumen SynCom, RuSynCom. a. Selection was based on 78 metagenomes from the rumen of cows. **b.** Phylogenomic tree of RuSynCom members with strain identifiers. **c**. Selection prevalence across the 78 samples and the Pfams captured by each member. **d**. Pfams accounted for at each stage of SynCom design. **e**. Number of mismatching Pfams encoded at each stage of SynCom design.

Figure S7: Comparison of SynComs for the mouse gut. a. The Pfams accounted for, and mismatches between both SynComs and 1,000 metagenomes from the mouse gut. **b.** Metabolic modelling of the two communities over seven hours. **c.** Pairwise interactions between the members of both SynComs were grouped into pre-defined categories and the frequency of these interactions plotted.

Figure S8: Initial selection of IBD and nonIBD SynComs based on a MAG collection. Each dot on the volcano plot represents a MAG which was selected to be part of at least one samples initial SynCom selection. Dots are coloured based on the phyla they belong to.

Figure S9: Identification of health-associated strains. a. Prevalence of each strain's selection within control samples across healthy cohorts. Each dataset is labelled based on the disease it focused on, the authors name, and year of publication. **b.** The most frequently selected strain, *Hungatella hathewayi* CLA-AA-H226, contained 499 Pfams that were consistently enriched within healthy control samples compared to their diseased counterparts. **c.** For each strain, the number of enriched functions within healthy samples, as both a percentage and count was plotted against the strains selection prevalence across healthy samples.

Table S1: Core and differentially present functions between studied populations. Lists containing the Pfam ID of each function identified as either core to a population, or differentially present in the pairwise comparison.

Table S2: Evaluation of the impact of functional weighting between 0 – 1 in increments of 0.0025. The impact of incrementally increasing the weighting applied in each of the three strategies to the 60 samples studied.

Table S3: Evaluation of the impact of functional weighting between 0 – 0.01 in increments of 0.001. The impact of incrementally increasing the weighting applied in each of the three strategies was applied to the 60 samples studied.

Table S4: Functional redundancy within HuSynCom. The number of functions encoded by subsets of HuSynCom are provided as absolute numbers, cumulative, and a cumulative percentage.

Table S5: Enrichment of protein families (Pfam) in IBD and nonIBD samples. The differential prevalence of each Pfam between the IBD and nonIBD samples as well as the p-value (Fischer exact test) are provided.

Table S6: MAG-based prediction of disease-specific SynComs. For each IBD and nonIBD sample, the sample-specific MAG-SynCom selections are provided, as well as taxonomic summaries to identify taxa enriched during selection.

Table S7: Overview of human gut bacterial isolates used during SynCom selection. Each strain, as well as their taxonomic assignment, is provided for both the initial collection of bacterial isolates, as well as the amended collection enhanced with the inclusion of additional *Pseudomonadota*.

Table S8: Overview of SynComs. For each SynCom studied experimentally within this manuscript, we provide their strain identifier, DSMZ deposition number, and StrainInfo identifier.

Table S9: Host parameters from IL10$^{-/-}$ mice colonised with the IBD and nonIBD SynComs. Data for the histopathological scoring, faecal lipocalin-2 measurements, and gene expression within the colonic tissue are provided.

Table S10: Overview of the SynCom selection process. The number of genomes and combinations of genomes being considered during each stage of MiMiC2 selection are stated.
